# Pennogenin Tetraglycoside Induces Rat Myometrial Contraction and MLC20 Phosphorylation via PLC-IP_3_ and RhoA/Rho Kinase Signaling Pathways

**DOI:** 10.1371/journal.pone.0051536

**Published:** 2012-12-12

**Authors:** Limei Wang, Chao Jia, Zuyin Yu, Xiaolan Liu, Liping Kang, Yue Cong, Yajun Shan, Zhenhu Zhao, Baiping Ma, Yuwen Cong

**Affiliations:** 1 Department of Pathophysiology, Beijing Institute of Radiation Medicine, Beijing, China; 2 Department of Biotechnology, Beijing Institute of Radiation Medicine, Beijing, China; Medical College of Wisconsin, United States of America

## Abstract

**Background:**

Total steroidal saponins extracted from the rhizome of *Paris polyphylla* Sm. var. *yunnanensis* (TSSPs) have been widely used in China for the treatment of abnormal uterine bleeding. We previously studied the main active constituents of TSSPs and their structure-activity relationships with respect to rat myometrial contractions. Tg (pennogenin tetraglycoside) was identified as one of the active ingredients in TSSPs able to induce rat myometrial contractions. However, the mechanisms underlying the pharmacological actions on uterine activity have not been described clearly.

**Methods:**

Here Tg was screened for effects on contractile activity in isolated uterine strips from estrogen-primed rats and on MLC20 phosphorylation and related signaling pathways in cultured rat myometrial cells as determined by Western blot. Intracellular calcium ([Ca^2+^]_i_) was monitored under a confocal microscope using Fluo-4 AM-loaded myometrial cells.

**Results:**

Tg dose-dependently stimulated rat myometrial contractions as well as MLC20 phosphorylation *in vitro*, which could be completely suppressed by an inhibitor of myosin light chain kinase (MLCK). Use of Ca^2+^ channel blockers and kinase inhibitors demonstrated that Tg-induced myometrial contractions are mediated by activation of the phospholipase C (PLC)-inositol triphosphate (IP3) signaling pathway, resulting in increased MLC20 phosphorylation. Furthermore, Y27632, a specific inhibitor of Rho kinase (ROK), notably suppressed Tg-stimulated myometrial contractions and decreased MLC20 phosphorylation.

**Conclusions:**

These data provide evidence that rat myometrial contractility induced by Tg results from enhanced MLC20 phosphorylation, while both PLC-IP3 and RhoA/ROK signaling pathways mediate the process. These mechanisms may be responsible for the therapeutic effects of TSSPs on abnormal uterine bleeding.

## Introduction

Gongxuening capsule (GXN) based on the yuannanbaiyao formulation, is a single preparation of total steroidal saponins prepared from *Paris polyphylla* Sm. var. *yunnanensis* (TSSPs). Steroidal saponins from the rhizome of *Paris polyphylla* var. *yunnanensis* have been isolated and studied by several groups [1], [2], [3], and the total steroidal saponins (GXN) have demonstrated reliable curative rates in the treatment of abnormal uterine bleeding (AUB), which can be attributed to its uterine contractile effects [4]. Due to its low cost, convenience and low incidence of side effects, GXN has been widely used in China for the treatment of AUB [5].

In our previous study, the strengthening of uterine contraction and promotion of hemostasis were found to be responsible for the therapeutic effects of GXN on AUB [6], [7]. Furthermore, based on other work in which TSSPs were isolated and identified [8], we constructed a compound library composed of a series of steroidal saponins purified from *Paris polyphylla* Smith var. *yunnanensis Paris polyphylla* Smith var. *yunnanensis* and steroidal saponins with similar structure using a varity of chemical methods. The chemical foundation of the steroidal saponins was then investigated by activity screening and analysis of structure-activity relationships [9]. Using bioassay-guided separation, the spirostanol-type steroidal saponins induced contractile activity in the myometrium, and several pennogenin glycosides were further purified and identified to be the active ingredients of TSSPs. Pennogenin tetraglycoside (Tg), one of the pennogenin glycosides with a spirostanol structure purified from TSSPs, was used as a probe to explore the signal transduction pathway underlying platelet aggregation, and its ability to stimulate secretion-dependent activation of rat platelets has been identified [10].

Although we have defined the general treatment effects of TSSPs on AUB and investigated to some extent the structure-activity relationship and possible function via activation of platelets, the exact mechanisms of the pharmacological actions, especially the signaling transduction pathways, on uterine contractions are still unclear.

MLC20, also known as “regulatory light chain”, has a pivotal role in regulating muscle contraction in vascular and uterine smooth muscles (SM) [11], [12]. Phosphorylation of Ser^19^ of MLC20 has been the primary interest in studies of regulation of SM contractile activity. This phosphorylation reaction can be mediated by MLCK, which is predominantly regulated by the concentration of free calcium ions (Ca^2+^) and the presence of calmodulin (CaM) [13]. Additionally, Rho kinase (ROK) can phosphorylate MLC20 directly or modulate it indirectly by phosphorylating the myosin phosphatase to reduce its activity [14]. However, previous studies have suggested that activation of SM contractions by agonists occur independently of MLC20 phosphorylation through myosin-binding activity but involve stimulation of the myosin ATPase activity [15], [16]. Therefore, in the present study, the role of MLC20 phosphorylation in Tg-induced myometrial contraction was first examined, and related pathways were further investigated. The overall aim of this study was to investigate the signaling transduction pathways involved in Tg-mediated induction of uterine myometrial contractions. Understanding the underlying mechanisms will facilitate discovery of the molecular targets of steroidal saponins in future drug development for AUB.

## Materials and Methods

### Materials

**Figure 1:**
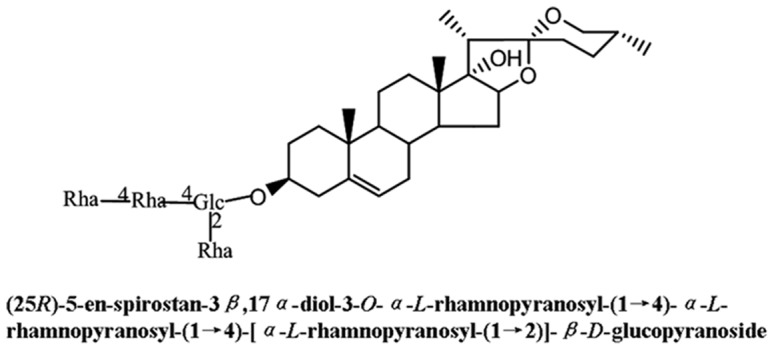
Chemical structure of Tg.

Chemicals used in the study, 2-aminoethoxydiphenyl borate (2-APB), ML-7, W-7, U73122, thapsigargin and Y27632, were purchased from Sigma (St. Louis. MO). Stock solutions of these inhibitors were prepared in dimethylsulfoxide (DMSO). Myosin light chain-2 antibody, phospho-myosin light chain-2 (ser19) antibody and horseradish peroxidase (HRP)-conjugated anti-rabbit IgG were obtained from Cell Signaling Technology (Beverly, MA). Tg was isolated from the TSSPs, and dissolved in DMSO [9]. The chemical structure of Tg is shown in Figure 1.

### Ethics Statement

Animal welfare and experimental procedures were carried out in accordance with the National Institutes of Health guidelines for the care and use of laboratory animals. This study was approved by Beijing Experimental Animal Ethics Committee (2006) No. 5118 set by the Beijing People's Government. Animals in this study were sacrificed by cervical dislocation. All efforts were made to minimize discomfort, distress, pain and injury.

### Animals

All experiments were performed with female Sprague Dawley (SD) rats, weighing 120–140 g, from the Laboratory Animal Center, Chinese Academy of Medical Sciences. The animals were kept in cages at 22±2°C with free access to pellet food and water on a 12-h light/dark cycle.

### Equipment

The rotary shaker (TS-2000), capillary electrophoresis system (DYY-5) and RJ-TDL-5A centrifuge were purchased from Ruijiang Instruments Co., Ltd (WuXi, China). The tension transducer connected to a polygraph system was purchased from Beijing Microsignalstar Techology Development Co., Ltd (Beijing, China).

### Assessment of Uterine Contractility *in vitro*

The animals were pretreated intraperitoneally with estradiol benzoate (0.1 mg/kg) for 2 days before the experiments. Rats were sacrificed by cervical dislocation. Uteri were excised, cleaned of adhering fat and mesentery tissues, and then cut into 10×2×2 mm^3^ strips along the longitudinal axis. The uterine strips were suspended vertically in 5-ml organ baths of Krebs’ solution (136 mM NaCl, 2.68 mM KCl, 1.8 mM CaCl_2_, 0.5 mM MgCl_2_, 11.9 mM NaHCO_3_, 0.32 mM NaH_2_PO_4_ and 5.04 mM glucose, pH 7.2), aerated continuously with 95% O_2_/5% CO_2_ and maintained at 37±0.2°C. Muscle tension was recorded isometrically with a tension transducer connected to a polygraph system. The preload condition was 1.0 g. The solution for each strip was first changed to 40 mM K^+^ for 10 min to ensure contractile viability and to determine maximum contraction, the recorded value of which was taken as the control [17]. Those strips that did not respond to KC1 were discarded. The equilibration period was not less than 30 min, and then spontaneous uterine contractions measured after the amplitude became stable were taken as the basal value. Subsequently, Tg was added to the bath solution to induce contractions. Subsequently, various inhibitors were added at 10 min intervals in a cumulative manner. The response curve of each uterine strip tested was plotted, and the contractions were represented by the area under the curve (AUC). Mechanical responses of uterine strips were analyzed as the area during a 10-min period after application of Tg or inhibitors and expressed as a percentage of the control.

### Culture of Dispersed Myometrial Cells

Myometrial SM cells were isolated from estrostilben primed rats. After the uteri were removed and dissected free of fat and endometrium, the tissues were cut into 1×1×1 mm^3^ pieces and placed in culture flasks containing DMEM medium supplemented with 15% fetal bovine serum (FBS) and maintained at 37°C in 5% CO_2_ atmosphere. Cells were subcultured every 3–4 days prior to reaching confluence. The semi-dispersed tissues were washed with PBS twice, and trypsin (0.25% w/v) was added. When most of the cells had contracted and become rounded, FBS was added to neutralize the effect, and then the suspension was centrifuged. The cells were resuspended with DMEM medium containing 10% FBS and plated on glass coverslips for 24 h at 37°C in 5% CO_2_ atmosphere before the experiments.

### Measurement of Changes in Intracellular Calcium

To assess the [Ca^2+^]_i_, the myometrial smooth muscle cells were loaded with the fluorescent calcium indicator by incubation of the cells in Fluo 4-AM (5 µmol/l, 37°C, Dojindo Laboratories, Japan) plus Pluronic F-127 for 60 min and Hanks’ balanced salt solution (PH = 7.4) alone for an additional 30 min to allow de-esterification of the dye. Changes in cytosolic free calcium concentration were monitored by detecting changes in fluorescence of single myometrial cells under a confocal scanning laser microscope (PerkinElmer UltraVIEW® VoX,USA). Fluo 4- AM was excited with the 494 nm line of an argon ion laser, and the emitted fluorescence was measured at 510 nm. A single myometrial cell was exposed to 2.5 µM Tg or 0.1% DMSO (vehicle control) for 120 s and the fluorescence was continuously recorded using time course software (Volocity Demo, USA).

### Total Protein Extraction

For Western blot analysis, myometrial cells were plated at a cell density of 5×10^4^ cells/well in 60-mm culture plates. After the cells had adhered to the plate, they were cultured in serum-free DMEM for 24 h before treatment with varying concentrations of Tg or other chemicals according to the experimental design. At the end of the desired treatment times, myometrial cells were washed with cold PBS three times, and then 2× SDS loading buffer was added. The cell lysates were then boiled for 10 min before centrifugation (10,000 g, 5 min). The samples were aliquoted and stored at −20°C for further use.

### MLC20 Phosphorylation Analysis

MLC20 phosphorylation was analyzed by Western blot as described previously [18]. In brief, the cell lysates were clarified by centrifugation at 12,000×*g* for 10 min at 4°C. Equal amounts of protein (20 µg) were separated through 12% SDS-PAGE and transferred to nitrocellulose membranes. The membranes were probed with specific primary antibodies, followed by an HRP-conjugated secondary antibody. Bound antibodies were detected with an enhanced chemiluminescence system detection kit (Cell Signaling). The change in MLC20 phosphorylation level after stimulation was monitored by using a phospho-Ser-19-specific antibody recognizing the phosphorylated MLC20 in the extracted protein solution.

### Statistical Analysis

Results are given as the mean ± S.E.M. for *n* samples. Statistical significance between means was determined using the Student’s t-test for two groups, and ANOVA was used for the means of multiple groups. *P* values <0.05 were considered significant.

## Results

### Pennogenin Tetraglycoside (Tg) Dose- and Time-dependently Induces Rat Uterine Contractions and MLC20 Phosphorylation in Rat Myometrial Cells

**Figure 2:**
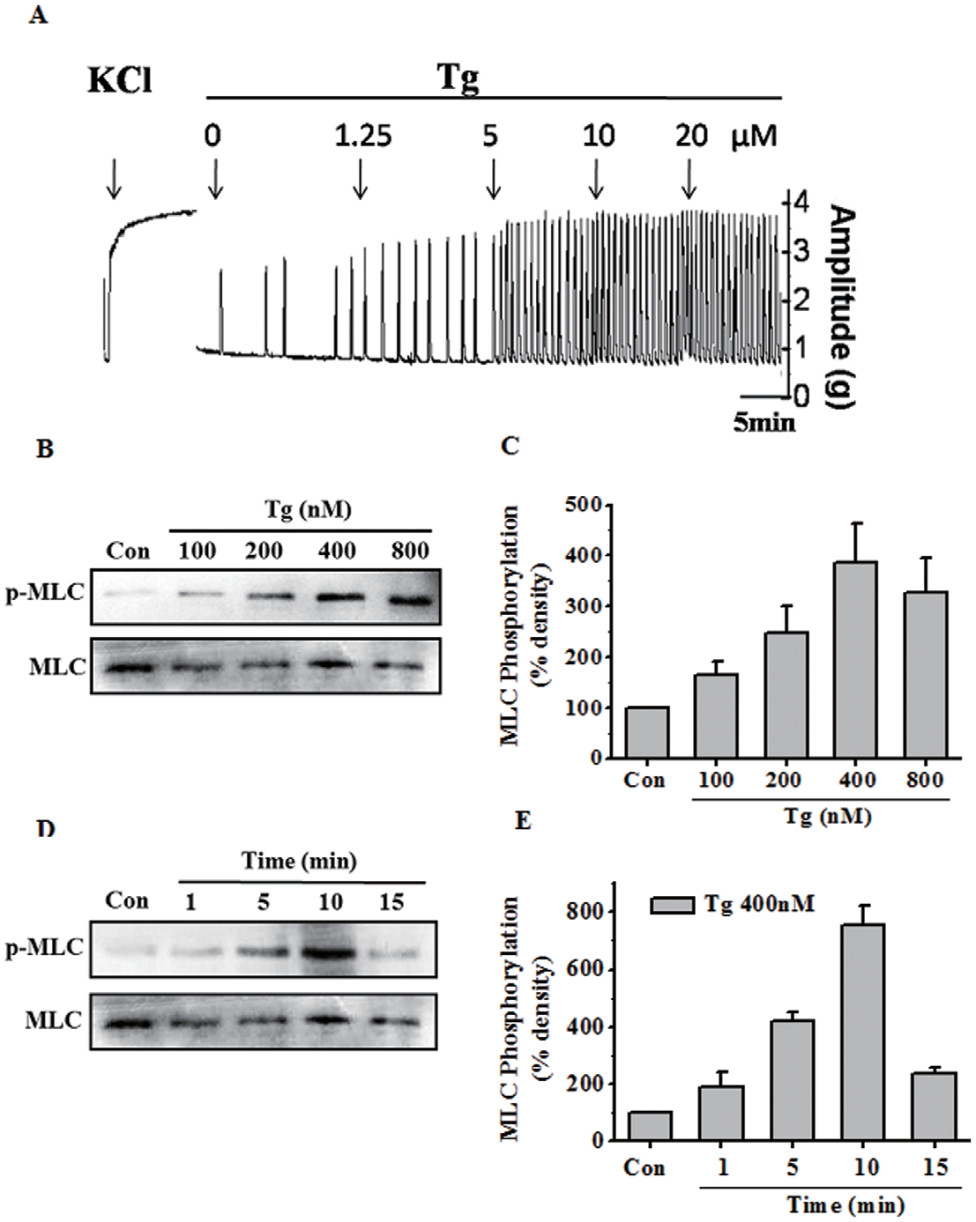
Tg dose- and time-dependently induced contractions of rat uterine strips and MLC20 phosphorylation in rat myometrial cells. (**A**) Representative recording of rat myometrial contractions induced by cumulative doses of Tg. Muscle tension was recorded isometrically with a tension transducer connected to a polygraph system. The solution for each strip was first changed to 40 mM K^+^ for 10 min to ensure contractile viability and to determine maximum contraction. (**B–E**) Representative antibody reaction blots for the relative levels of MLC20 and pMLC20 in protein samples from rat myometrial cells treated with cumulative doses of Tg (B) or 400 nM Tg for different period of time (D). Quantitative analyses of the pMLC20-to-MLC20 ratio (C, E). Signal intensities for MLC20 and pMLC20 from three different blots were used for the quantitative analyses. Data are expressed as means ± SEM.

Our previous study showed that TSSPs can induce rat myometrial contractions, and the components with a spirostanol stucture displayed the strongest contractile activities. In the current work, we found that Tg, a spirostanol-type compound from the extract of TSSPs (Figure 1), could also dose-dependently induce myometrial contractions (Figure 2A), even at a very low concentration (1.25 µM).

Several concentrations of Tg were tested to determine its effect on MLC20 phosphorylation. As shown in Figure 2B and 2C, the application of Tg for 10 min induced MLC20 phosphorylation in a dose-dependent manner. Subsequently, Tg at 400 nM was applied for different response times, and the Western blot analysis of phosphorylated MLC20 showed a typical time-dependent effect (Figure 2D and 2E). These data indicated that Tg can dose- and time-dependently induce rat uterine myometrial contractions, and the effect may be related to MLC20 phosphorylation.

### Antagonist of MLCK can Inhibit MLC20 Phosphorylation and Uterine Contractions Induced by Tg

**Figure 3:**
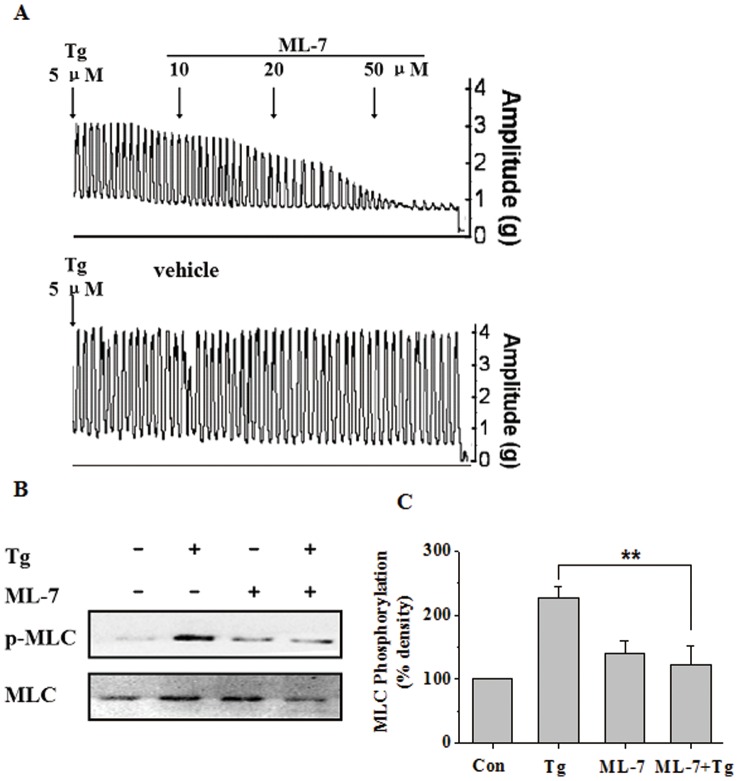
MLCK is involved in Tg-induced MLC20 phosphorylation and uterine contractions. (**A**) Representative recording of the inhibitory effects of cumulative doses of MLCK inhibitor ML-7 on rat myometrial contractions induced by 5 µM Tg, while the control strips were added with vehicle at the same time after stimulation with Tg. (**B–C**) Representative antibody reaction blots for the relative levels of MLC20 and pMLC20 in protein samples from Tg (400 nM) -treated or untreated rat myometrial cells in the presence or absence of ML-7 (500 nM), a specific inhibitor of MLCK (B). Signal intensities for MLC20 and pMLC20 from three different blots were used for the quantitative analyses (C). Data are expressed as means ± SEM. ** *P*<0.01 compared to control.

The regulation of MLC20 phosphorylation is known to be mediated by the enzyme MLC20 kinase (MLCK), which is predominantly regulated by the intracellular concentration of free calcium ion ([Ca^2+^]i). Considering the involvement of MLCK in myometrial contractions and Tg-induced MLC20 phosphorylation, a specific MLCK inhibitor, ML-7, was used to determine the mechanism of Tg-induced myometrial contractions in the following experiments. As shown in Figure 3A, treatment of myometrial strips with 5 µM Tg caused rhythmic contractions. Exposure to different concentrations (10, 20, 50 µM) of ML-7 significantly inhibited Tg-induced myometrial contractions when compared with the control. At the concentration of 50 µM, ML-7 completely inhibited the rhythmic contractions. The inhibition by ML-7 involved a significant reduction of the amplitude but not the frequency of myometrial contractions, suggesting that MLCK activity is essential for the Tg-induced production of contractile mechanical force. The Tg-induced MLC20 phosphorylation in the presence of ML-7 was also tested. As shown in Figure 3B and 3C, ML-7 significantly inhibited MLC20 phosphorylation induced by Tg (*P*<0.01). Overall, these results support an essential role for MLCK in Tg-stimulated MLC20 phosphorylation during uterine contractions.

### Involvment of [Ca^2+^]_i_ Increase and CaM Activity in Tg-induced Uterine Contractions and MLC20 Phosphorylation

**Figure 4:**
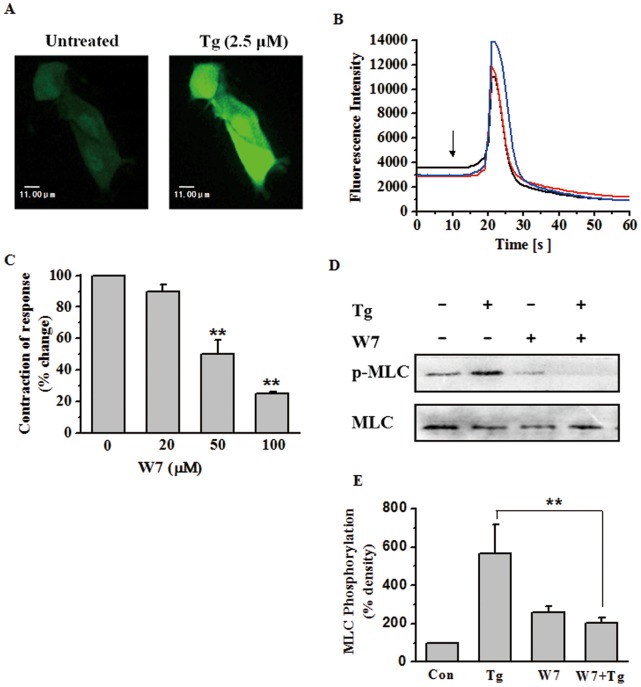
Involvment of [Ca^2+^]_i_ increase and CaM activity in Tg-induced uterine contractions and MLC20 phosphorylation. (A) Confocal fluorescence images of myometrial smooth muscle cells loaded with Fluo-4 AM before and after 2.5 µM Tg stimulation. The fluorescence images were observed at the excitation wavelength of 510 nm and 12 s after Tg stimulation. (B) Time course of the change of the fluorescence in a Fluo-4 AM-loaded myometrial smooth muscle cells in response to Tg (2.5 µM). The arrow indicates the time at which Tg was added. (**C**) Inhibitory effects of cumulative doses of W-7 on contractile responses after stimulation with Tg in rat myometrial strips (n = 3). Contractions were measured as the area under the curve (AUC) and expressed as a percentage of the response to 5 µM Tg. (**D–E**) Representative antibody reaction blots for the relative levels of MLC20 and pMLC20 in protein samples from Tg (400 nM) -treated or untreated rat myometrial cells in the presence or absence of W-7 (500 nM), a common antagonist of calmodulin (D). Signal intensities for MLC20 and pMLC20 from three different blots were used for the quantitative analyses (E). Data are expressed as means ± SEM. ** *P*<0.01 compared to control.

Phosphorylation of MLC20 in rat myometrial cells can be mediated by MLCK, which is predominantly regulated by the concentration of free calcium ions (Ca^2+^) and the presence of calmodulin (CaM). The alterations in [Ca^2+^]_i_ in response to Tg were then detected in the Flu-4-AM loaded myometrial cells. Flu-4-AM, a cell-permeable acetoxymethyl ester, produces a large increase of fluorescence intensity upon binding Ca^2+^. As shown in Figure 4A, the myometrial cells loaded with Flu-4 AM exhibited a transient significant increase in fluorescence after stimulation with 2.5 µM Tg. As a control, cells treated with vehicle did not show any significant change in fluorescence (data not shown). In the same experiments, while continuously recording the fluorescence using a time course software, a rapid transient peak of fluorescence in myometrial cells was observed when challenged with Tg (Figure 4B), which indicated an increase in [Ca^2+^]_i_ in rat myometrial cells. Furthermore, the increase of fluorescence was not reversed for at least 5 min, and retreatment with Tg did not produce an additional peak in the fluorescence (data not shown).

Calmodulin (CaM) is a calcium-binding messenger protein expressed in myometrial cells and CaM-dependent myosin light chain kinase (MLCK) is considered the primary regulator of MLC20 phosphorylation. To investigate the role of CaM in Tg-induced myometrial contractions and MLC20 phosphorylation, W-7 was used to prevent CaM from binding target proteins. In the contraction experiments, W-7 was applied at concentrations of 0, 20, 100 and 500 µM, while a single concentration of 500 nM was chosen for the Western blot analysis. W-7 could strongly inhibit the Tg-induced myometrial contractions (*P*<0.01, Figure 4C) in a dose-dependent manner as well as decrease the level of MLC20 phosphorylation (*P*<0.01, Figure 4D and 4E). These data suggest that CaM is integral to the mechanism of Tg-induced myometrial contractions and MLC20 phosphorylation by regulating the activity of MLCK.

### Role of Extracellular as Well as Intracellular Calcium in Tg-induced Uterine Contractions and MLC20 Phosphorylation

**Figure 5:**
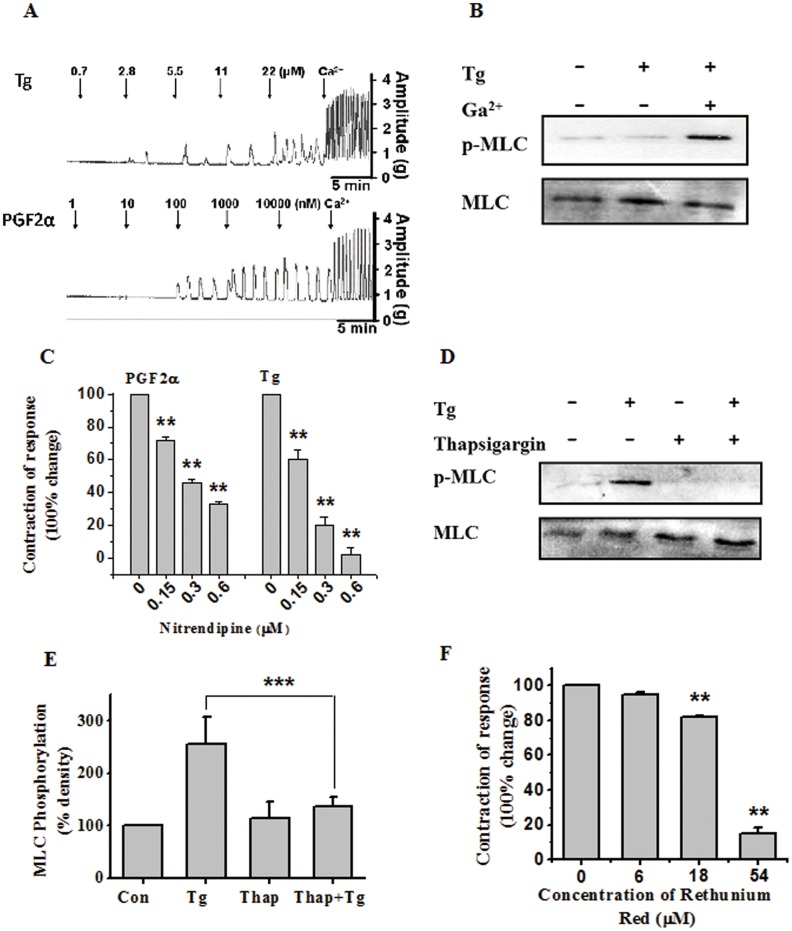
Role of extracellular as well as intracellular calcium in Tg-induced uterine contractions and MLC20 phosphorylation. (**A**) Representative recordings of the cumulative dose responses on myometrial strips induced by Tg or PGF-2α in calcium-free solution. After the stimulators were increased to the maximal effective dose, calcium was added to a final concentration of 1.8 mmol/L. (**B**) Representative antibody reaction blots for the relative levels of MLC20 and pMLC20 in protein samples from Tg (400 nM) -treated or untreated rat myometrial cells in the presence or absence of extracellular Ca^2+^. (**C**) Inhibitory effects of cumulative doses of nitrendipine on contractile responses after stimulation with Tg or PGF-2α in rat myometrial strips (n = 3). Contractions were measured as the area under the curve (AUC) and expressed as a percentage of the response to 5 µM Tg or 450 nM PGF-2α. (**D–E**) Representative antibody reaction blots for the relative levels of MLC20 and pMLC20 in protein samples from Tg (400 nM) -treated or untreated rat myometrial cells in the presence or absence of thapsigargin (2.5 µM) (D). Signal intensities for MLC20 and pMLC20 from three different blots were used for the quantitative analyses (E). (**F**) Inhibitory effects of cumulative doses of Rethenium red on contractile responses after stimulation with Tg in rat myometrial strips (n = 3). Contractions were measured as the area under the curve (AUC) and expressed as a percentage of the response to 5 µM Tg. Data are expressed as means ± SEM. *** *P*<0.001 and ** *P*<0.01 compared to control.

The roles of extracellular as well as intracellular Ca^2+^ were investigated in subsequent experiments. As shown in Figure 5A, spontaneous oscillatory uterine contractions were abolished in the absence of extracellular Ca^2+^. Addition of Tg or PGF-2α (as a positive control) did not restore contractility in Ca^2+^-free buffer, but resulted in some contraction waves at high concentrations. However, when CaCl_2_ was subsequently added at the physiological concentration, phasic contractions resumed in Tg or PGF-2α pretreated myometrial strips. The role of extracellular Ca^2+^ in Tg-induced MLC20 phosphorylation was also examined (Figure 5B). The results indicate that Tg can not induce myometrial MLC20 phosphorylation without the influx of extracellular Ca^2+^, but upon *addition* of Ca^2+^, MLC20 was strongly *phosphorylated. In Addition,* only presence of extracellular Ca^2+^ without the stimulation of Tg also can not induce MLC20 phosphorylation (data not shown). The effect of nitrendipine, a voltage-operated L-type calcium channel blocker, which inhibits the influx of extra cellular calcium [19], on Tg-stimulated uterine contractions was then determined. As shown in Figure 5C, nitrendipine dose-dependently reduced both the amplitude and frequency of Tg as well as PGF2α stimulated myometrial contractions, resulting in significant decreases in contractile activity. When the concentration of nitrendipine was increased to 0.6 µM, oscillation of uterine contractions induced by Tg was completely abolished.

Thapsigargin was also used to investigate the role of [Ca^2+^]_i_ in Tg-induced MLC20 phosphorylation. Thapsigargin, a non-competitive inhibitor of the sarco/endoplasmic reticulum Ca^2+^ ATPase (SERCA) class of enzymes [20], raises cytosolic Ca^2+^ concentrations by blocking the ability of the cell to pump the ion into the sarcoplasmic and endoplasmic reticula, causing these stores to become depleted. As shown in Figure 5D and 5E, the application of thapsigargin could significantly inhibit MLC20 phosphorylation induced by Tg (*P*<0.01). Furthermore, ruthenium red, a ryanodine receptor inhibitor which inhibits Ca^2+^-induced Ca^2+^ release from the ryanodine-sensitive intracellular stores [21], also significantly inhibited Tg-induced phasic contractions over a concentration range of 5–55 µM (*P*<0.01) (Figure 5E).

Hence, it was proposed that the influx of Ca^2+^ through voltage-operated Ca^2+^ channels may play an important role in Tg-induced uterine contractions. During Tg-induced myometrial contractions, the release of intracellular Ca^2+^ also plays an essential role. The increase in [Ca^2+^]_i_ is known to result in formation of the Ca^2+^-CaM complex in myometrial cells, which activates muscle MLCK. The subsequent phosphorylation of regulatory myosin light chains allows them to rapidly bind to and detach from actin filaments, thereby generating tension [22].

### PLC-IP3 Signaling Pathway is Involved in Tg-induced Myometrial Contractions through the Regulation of [Ca^2+^]_i_

**Figure 6:**
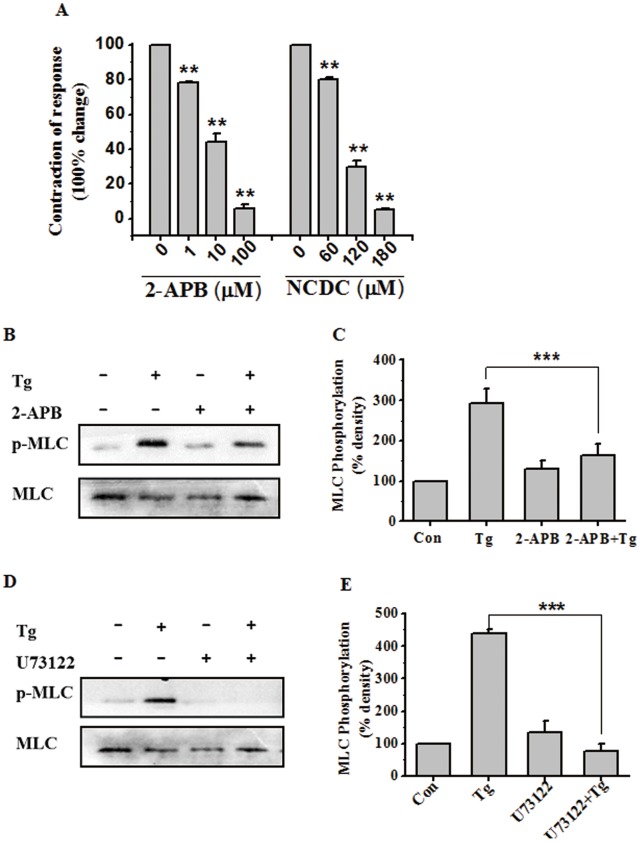
PLC-IP3 signaling pathway is involved in Tg-induced myometrial contractions through the regulation of [Ca^2+^]_i_. (**A**) Inhibitory effects of cumulative doses of 2-APB and NCDC on contractile responses after stimulation with Tg in rat myometrial strips (n = 3). Contractions were measured as the area under the curve (AUC) and expressed as a percentage of the response to 5 µM Tg. (**B–E**) Representative antibody reaction blots for the relative levels of MLC20 and pMLC20 in protein samples from Tg (400 nM) -treated or untreated rat myometrial cells in the presence or absence of 2-APB (2-APB inhibitor) (B) or U73122 (PLCβ inhibitor) (D). Signal intensities for MLC20 and pMLC20 from three different blots were used for the quantitative analyses (C, E). Data are expressed as means ± SEM. *** *P*<0.001 compared to control.

Previous reports have demonstrated that phosphoinositide-specific PLC (PI-PLC) is an important component of the myometrial intracellular oscillator. PLC hydrolyzes PIP2 to yield IP3, which binds specific receptors on the endoplasmic reticulum (ER) to mobilize Ca^2+^ from internal stores [23]. 2-APB, a novel membrane-permeable IP3-receptor inhibitor [24], significantly inhibited phasic myometrial contractions stimulated with Tg (*P*<0.01) (Figure 6A). In addition, inhibition of PI-PLC with 2-nitro-4-carboxyphenyl-N, N-diphenylcarbamate (NCDC), a PLC specific inhibitor, also significantly inhibited phasic myometrial contractions stimulated by Tg, further supporting the key role of IP3 production in the contractile process (Figure 6A). By Western blot analysis, both 2-APB (blocks IP3 receptors which would otherwise cause release of Ca^2+^) and U73122 (inhibitor of PLCβ, directly inactivates PLCβ) could significantly suppress Tg-induced MLC20 phosphorylation (*P*<0.01) (Figure 6B–E). These results demonstrate that the PLC-IP3 pathway plays an essential role in Tg-stimulated rat myometrial contractions by regulating MLC20 phosphorylation.

### RhoA/ROK Signaling Pathway Mediates Tg-induced Myometrial Contractions via Regulating MLC20 Phosphorylation

**Figure 7:**
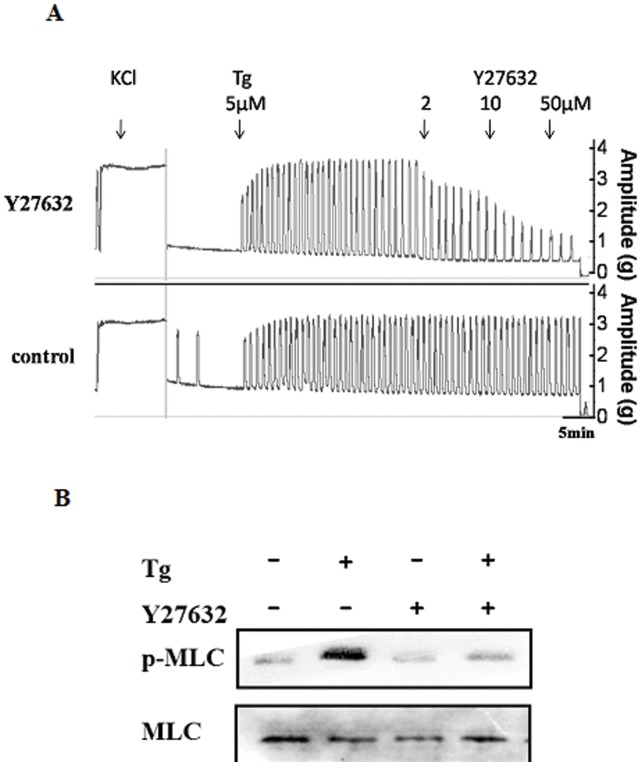
RhoA/ROK signaling pathway mediates Tg-induced myometrial contractions via regulating MLC20 phosphorylation. (A) Inhibitory effects of cumulative doses of Y27632 on contractile responses after stimulation with Tg in rat myometrial strips (n = 3). The control strips were added with vehicle at the same time after stimulation with Tg (5 µM). (B) Representative antibody reaction blots for the relative levels of MLC20 and pMLC20 in protein samples from Tg (400 nM) -treated or untreated rat myometrial cells in the presence or absence of Y27632.

RhoA is a small monomeric G-protein and a member of the Rho subfamily of the Ras superfamily of monomeric GTPases [25]. ROK, a serine/threonine kinase [26], [27], is one of the main signal transduction effectors of RhoA. Previous studies have suggested that RhoA and ROK are involved in agonist-induced uterine myometrial contractions [28], [29]. The ROK inhibitor Y27632 was used to determine whether the RhoA/ROK pathway is involved in Tg-induced MLC20 phosphorylation resulting in myometrial contractions. As shown in Figure 7A, Y27632 could inhibit contractions induced by Tg at 5 µM and even at 2 µM, with nearly complete suppression at 50 µM. In the Western blot analysis (Figure 7B), significant inhibition of MLC20 phosphorylation induced by 500 nM Tg was observed.

## Discussion

Steroidal saponins are widely distributed in the botanical kingdom, possessing a broad range of biological and pharmacological properties, such as hypocholesterolemic, anti-tumor, anti-diabetic, anti-inflammatory, anti-fungal and platelet agonistic and inhibitory activities [30]. In our previous studies, we analyzed the structure-activity relationships of a series of spirostanol glycosides isolated from *Paris polyphylla* Smith var. *yunnanensis* in uterine contractions [9]. In this study, we chose Tg as a typical extract with a spirostanol structure purified from TSSPs and focused on the mechanisms underlying its activities. Our results led to three major implications or conclusions. First, the active compound Tg significantly stimulated phasic contractions of rat myometrial strips in a concentration-dependent manner. Meanwhile, application of Tg to cultured rat myometrial cells could dose and time-dependently induce MLC20 phosphorylation. In view of these results, we believe that Tg-induced rat myometrial contraction is related to the phosphorylation of MLC20, which has a pivotal role in regulating muscle contraction in vascular and uterine smooth muscle [11], [12]. Furthermore, experiments with Ca^2+^ channel blockers and several kinase inhibitors demonstrated that Tg-induced myometrial contractions involve activation of the PLC-IP3 signaling pathway, resulting in MLC20 phosphorylation. Finally and importantly, the Tg-induced myometrial contractions and MLC20 phosphorylation could be inhibited by Y27632, a specific ROK inhibitor, suggesting that the RhoA/ROK signaling pathway is also involved in the process. These mechanisms may be responsible for the therapeutic effect of TSSPs on AUB.

The term ‘myosin’ refers to a large superfamily of genes that share the ability to bind to actin and possess ATPase activity [31]. The ‘myosin motor’ of human muscle tissue is predominantly of the class myosin II (MII) [32]. In SM, MII is a hexamer molecule composed of two myosin heavy chains (MHC) and two pairs of myosin light chains (MLC). The two MLCs have molecular masses of 20 (MLC20) and 17 (MLC17) kDa. Phosphorylation of Ser19 on MLC20 causes a conformational change that increases the angle in the neck domain of the MHC, thus mobilizing the cross-bridges and causing the actin thin filament to slide along the myosin thick filament. As presented in this paper, we found that Tg could induce the phosphorylation of Ser19 on MLC20, which was suppressed by ML-7, an inhibitor of the MLC20 kinase (MLCK) which is predominantly regulated by [Ca^2+^]_i_. We also observed that ML-7 significantly reduced the amplitude but not the frequency of myometrial contractions induce by Tg, as MLC20 activity but not [Ca^2+^]_i_ signals was inhibited.

In common with other SM tissues, changes in [Ca^2+^]_i_ signals within the myometrium have important functional consequences, as they determine contractility [22], [33]. For uterine contractions, [Ca^2+^]_i_ can be increased by influx from the extracellular space, release from intracellular storage sites, or both [34]. CaM is a Ca^2+^-dependent cytosolic protein which binds four Ca^2+^ ions [35]. The 4 Ca^2+^-CaM complex activates the key enzyme MLCK, causing an immediate and marked increase in phosphorylation of MLC20, which activates the contractile machinery [36]. Interestingly, when using W-7, free Ca^2+^ ions and thapsigargin, which would affect the [Ca^2+^]_i_ of rat myometrial cells, significant inhibiton or enhancement of Tg-induced MLC20 phosphorylation was observed.

Previous reports have demonstrated that agonist-induced myometrial contractions are coupled to phosphoinositidespecific phospholipase C (PI-PLC) activation and inositol 1,4,5-trisphosphate (IP3) production. IP3 induces a rise in free cytosolic calcium via the release of intracellular calcium from IP3-sensitive stores and the calcium-induced calcium release [23]. Furthermore, 2-APB and U73122, which are inhibitors of IP3 and PLCβ, respectively, almost completely suppressed the Tg-induced MLC20 phosphorylation, also significantly reduced the amplitude as well as the frequency of myometrial contractions induce by Tg. These findings suggests that the PLC-IP3 signaling transduction pathway, which directly regulates the [Ca^2+^]_i,_ is extremely important in Tg-induced MLC20 phosphorylation and the resulting phasic myometrial contractions.

A variety of contractile agonists trigger activation of the small GTPase RhoA. An important target of activated RhoA in smooth muscle is Rho-associated kinase (ROK), one of the downstream targets that is the myosin binding subunit (MYPT1) of myosin light chain phosphatase (MLCP). ROK phosphorylates the myosin targeting subunit (MYPT1) of MLCP at two potential sites (T696, T853), thus results in a decrease in phosphatase activity of MLCP and an increase in myosin light chain phosphorylation catalyzed by Ca^2+^/calmodulin-dependent myosin light chain kinase. The RhoA/ROK pathway has been implicated in the tonic phase of force maintenance in response to various agonists, with no evident role in the phasic response [25]–[27]. As reported, oxytocin-stimulated contractions of human myometrium obtained at term elective caesarean sections are inhibited by the ROK inhibitor Y27632 independently of the change in [Ca^2+^]_i_ [37]. In our study, application of Y27632 could significantly inhibit MLC20 phosphorylation, as well as the amplitude but not the frequency of myometrial contractions induce by Tg. These results suggest that Tg can enhance MLC20 phosphorylation in myometrial cells and rat myometrial contractions via the RhoA/ROK pathway.

The intracellular PLC-IP3 signaling pathway is known to be activated by G-protein coupled receptor (GPCR) signaling, which is mediated by the G_q_ protein. Recent data demonstrate that G_α12/13_ can induce Rho-dependent responses. Furthermore, G_α12/13_ can bind and activate Rho-specific guanine nucleotide exchange factors, providing a mechanism by which GPCRs that couple to G_α12/13_ could activate Rho and its downstream responses [38]. Therefore, we boldly speculate that the target of Tg for inducing myometrial contractions may be a type of GPCR.

Steroidal saponins, present in plants and some marine animals, have many pharmacological and biological activities [30], [39]. Our group first reported that TSSPs are strong contractile stimulators for the uterus [7] and that pennogenin glycosides with a spirostanol structure are the active ingredients of TSSPs in promoting uterine myometrial contractions *in vivo* [9]. Here, we report that Tg, a spirostanol-type pennogenin glycoside and one of the active ingredients of TSSPs, is capable of inducing rat myometrial contractions, and these activation responses are dependent on MLC20 phosphorylation, which is regulated by both PLC-IP3 and RhoA/ROK signaling pathways. The involvement of corresponding GPCRs in the above process is highly likely. In fact, previous structure-activity assays showed that spirostanol glycosides exhibit structure-related inducible or inhibitory activity in rat uterine contractions, which are attributed not only in part to the number, length and position of sugar side chains attached to the glycoside but also to the structure of the aglycone. We inferred that the ultimate effects on specific receptors of the myometrial cells contribute to differences in contraction or relaxation responses. The main ingredients of GXN [4] has been proposed to strengthen uterine contractions and/or promote hemostasis *in vivo*, and pennogenin glycosides with a spirostanol structure may be responsible for the therapeutic effects on AUB. These mechanistic insights will be helpful for identifying molecular targets of steroidal saponins for the purpose of drug development in the treatment of AUB.

In summary, this study demonstrates that rat myometrial contractility induced by Tg results from MLC20 phosphorylation in myometrial cells, while the PI-PLC and RhoA/ROK signaling transduction pathways mediate the process. Furthermore, the two signaling pathways act under different mechanisms, one occurs via the changes of [Ca^2+^]_i_ regulated by PI-PLC pathway, while the other via Ca^2+^ sensitization of the contractile proteins signaled by the RhoA/Rho kinase pathway. We speculate that specific GPCRs may be targeted in the response to Tg, which may be responsible for the therapeutic effect of TSSPs on AUB. These findings may help facilitate the drug discovery process for AUB therapies.
